# Testing the evolutionary basis of the predictive adaptive response hypothesis in a preindustrial human population

**DOI:** 10.1093/emph/eot007

**Published:** 2013-04-18

**Authors:** Adam D. Hayward, Virpi Lummaa

**Affiliations:** Department of Animal and Plant Sciences, University of Sheffield, Western Bank, Sheffield S10 2TN, UK

**Keywords:** metabolic syndrome, silver spoon, human life-history, developmental constraint, developmental plasticity, early-life nutrition

## Abstract

**Background and objectives:** The thrifty phenotype hypothesis proposes that late-life metabolic diseases result from mismatch between early-life and adulthood nutrition. More recently, the predictive adaptive response (PAR) hypothesis has suggested that poor early-life environmental conditions induce metabolic changes that maximize health and fitness in similarly poor adult conditions, but reduce fitness if conditions later improve. Therefore, later-life survival and reproduction should be maximized where environmental conditions during development and adulthood match, but few studies in humans have addressed the consequences of poor early conditions on fitness traits in varying later conditions.

**Methodology:** We tested key evolutionary predictions of the PAR hypothesis using detailed longitudinal data with several environmental parameters from a natural fertility preindustrial human population, to investigate how combinations of early- and late-life environmental conditions affected annual probabilities of survival and reproduction.

**Results:** We found no suggestion that fitness was maximised when developmental and later-life conditions matched, but rather poor environmental conditions during development or later life and their combinations were associated with lower survival.

**Conclusions and implications:** Our results are more consistent with predictions of ‘silver spoon’ models, whereby adverse early-life conditions are detrimental to later health and fitness across all environments. Future evolutionary research on understanding metabolic disease epidemiology should focus on determining whether adaptive prediction maximizes infant survival where conditions match during development and immediately after birth, rather than drawing attention to the unlikely long-term fitness benefits of putative metabolic changes associated with poor early nutrition.

## Abstract (precis)

Mismatch between developmental and adulthood conditions is thought to lead to reduced fitness. We tested the evolutionary basis of this influential hypothesis in a preindustrial human population. However, we found no support for the key prediction that survival and reproduction would be maximised where developmental and later-life conditions match.

## BACKGROUND AND OBJECTIVES

It is well-established that nutritional and environmental conditions during development can have consequences for health and fitness in humans and wild animals [1, 2]. The adaptive significance of such effects has been appreciated among evolutionary ecologists and this evolutionary framework has increasingly been applied in order to understand human disease [3]. In humans, poor nutrition during gestation is linked to increased later-life risk of heart disease and type II diabetes [4], due to developmental changes in glucose metabolism, organ growth and vasculature [5]. The ‘thrifty phenotype’ hypothesis viewed this as an evolved response to poor nutrition during gestation, favoured by selection because it enhanced neonatal survival in low-nutrition environments, with later-life health problems the maladaptive consequence of improved nutrition [5]. Although the original hypothesis proposed that physiological plasticity maximizes foetal survival to the detriment of later-life health [4], it has recently been suggested that selection for such plasticity could act through enhancing reproductive performance and survival in adulthood [3, 6]. This ‘predictive adaptive response’ (PAR) hypothesis proposes that nutritional information alters foetal development to maximize fitness when later-life conditions match those around birth, but leads to lower fitness when a mismatch occurs [6, 7].

Determining the fitness consequences of matching and mismatching early and later environments is a key test of whether the PAR hypothesis best explains the epidemiology of metabolic diseases, but applications of this test are rare. Such studies would also broaden understanding of the evolutionary basis of adaptive developmental plasticity, since previous studies of early-life environmental effects on fitness rarely consider variable later-life conditions, and therefore cannot explore how early conditions interact with variable later-life conditions. This is essential for determining the adaptive nature, extent and costs of such prediction [8]. One exception is a recent study of a historical Canadian population, which investigated the interacting effects of birth season and migration between high- and low-quality habitats on survival. The optimal birth season for survival differed between habitats, but the advantage was lost if individuals switched habitat in later life [9]. Therefore, a match between early- and later-life conditions enhanced survival, although the study only considered effects on ages older than 60 years rather than reproductive-aged individuals, selection on whom is stronger. Another study on contemporary Polish women showed that oestrodiol (E2) levels (positively associated with conception probability) were higher among women who exhibited low levels of physical activity compared with those who exhibited high levels, and were independent of fatness at birth within activity levels [10]. However, at moderate activity levels, E2 levels were higher among women who were fat at birth than among those who were skinny, indicating that being skinny at birth was associated with reduced ability to maintain fertility with increasing activity [10]. These results do not support the PAR hypothesis, which would have predicted higher E2 at high activity levels among women skinny at birth than women fat at birth.

In summary, although the PAR hypothesis is a prominent explanation in the medical and epidemiological literature for the evolution of metabolic disease [3, 6], little attention has been paid by evolutionary biologists to the ways in which variable early and later environments could shape patterns of mortality and reproduction in long-lived species. Such an approach could resolve inconsistencies in the literature on the delayed effects of developmental environments on adult fitness, determining whether prediction of the adult environment during early life is likely to have played a role in human life-history evolution [8]. The validity of this hypothesis for the evolution of metabolic plasticity has important implications for understanding and predicting epidemiological patterns, as well as treatment and intervention [11].

We used a 127-year longitudinal dataset from four preindustrial Finnish agricultural populations to investigate fitness outcomes of variable environmental conditions during development and later life. These longitudinal church records monitor each individual from birth until death. In these natural mortality and fertility populations, we used three measures of environmental quality, allowing us to monitor the annual survival and reproduction of individuals in relation to annual variation in early- and later-life conditions. This may provide a better reflection of the conditions under which human life histories have evolved than famine cohort studies, such as that of the Dutch ‘hunger winter’ in 1944–45 [12] which only test the fitness consequences of varying birth environments in favourable later-life conditions. If PARs play a role in human development, we predict that individual survival or reproductive success should be higher in individuals where early-life conditions match conditions in later life, compared with where they do not match. For instance, where later-life conditions are poor, individuals who experienced poor conditions during development should experience higher fitness than individuals who experienced good conditions during development. Similarly, where later-life conditions are favourable, individuals developing in favourable conditions are expected to experience higher fitness than individuals who developed in poor conditions. Conversely, if prediction is not adaptive, then individuals who experience poor early-life conditions should have lower subsequent fitness compared with those experiencing favourable early-life conditions, irrespective of later-life conditions.

## METHODOLOGY

### Study populations

We investigated the interacting effects of early- and later-life environmental conditions on annual survival and reproduction in natural fertility human populations. We used church records collected from 1750 onwards to construct individual life histories across four rural Finnish ‘parishes’ (Hiittinen, Kustavi, Rymättylä and Ikaalinen) [13]. We monitored annual survival and reproduction of individuals 1751–1877, before improved healthcare and birth control reduced birth and death rates [14]. Forty-two percent of individuals died before age 15 years, consistent with patterns in contemporary hunter–gatherers [15]. Causes of death are recorded in parish registers, and the major causes among children were typhoid, smallpox, shigellosis and ‘respiratory disease’, whereas over 50% of adults died of typhoid, tuberculosis or ‘old age’. The mean age at first reproduction among women in our data was 25.93 ± 0.18 years, with reproductive females producing a mean of 4.65 ± 0.10 children, with a mean age at last reproduction of 35.96*y* ± 0.24. Among females who lived to age 15 years, the average lifespan was 57.56*y* ± 0.56, whereas among men, it was 55.78*y* ± 0.49. We included data from individuals who either had a recorded death date or who survived to 1877, the last year for which environmental records were available.

### Environmental data

**Figure 1.**
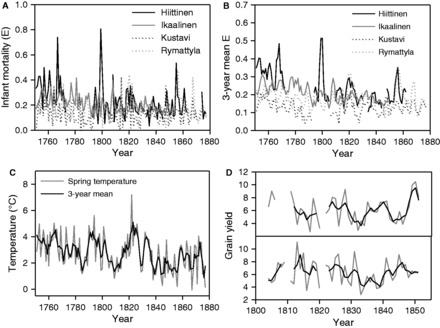
Variation in environmental conditions across the study period. The figure shows that all three measures of environmental conditions varied considerably across the study period (1751–1877). (**A**) The proportion of infants dying in their first year of life (*E*) in the four parishes varied between 0.00 and 0.81. (**B**) The 3-year means of *E* in the four parishes varied between 0.04 and 0.51. (**C**) Spring temperature varied substantially over the study period even after taking 3-year means. (**D**) Both the annual yields (grey lines) and 3-year mean yields (black lines) of rye (top) and barley (below) varied across the restricted study period over which we examined the survival of Ikaalinen children aged under 15 years.

We used three measures of inter-annual variation in environmental conditions (Fig. 1):

- Infant mortality (*E*): We used a demographic measure of environmental quality: the proportion of children born in a given year that died in their first year (*E*), as used in previous studies [16]. *E* was calculated as the proportion of infants born in each parish in a given year who died in their first year of life.
- Spring temperature: We used spring temperatures reconstructed using standard multiproxy techniques from historical data including sea ice break-up and plant phenology. These factors explained a large proportion of the variance in observed February–June temperatures in south-west Finland and were used to reconstruct spring temperatures from 1750 onwards [17]. This measure is associated with the severity of the winter [17] and is positively associated with the success of the critically important rye harvest [18], making it suitable for analyses of PAR hypotheses. Although temperatures at other times of year are potentially related to disease transmission, several studies in Finland have found only weak links between temperature and disease-related deaths [19, 20], with the strongest link being a positive associations between spring and summer temperatures and increased disease transmission through contaminated food and water and increased movement of people [21].
- Crop yields: Finally, we obtained data on local crop yields, reflecting inter-annual variation in harvest success [18], which is associated with mortality in this population [22]. These data were only available for Ikaalinen, and results of this analysis are shown in the Supplementary Information. Crop yields are calculated annually as the amount of grain harvested as a multiple of that sown.

We investigated the effects of early- and later-life environmental conditions on individual annual mortality and reproduction. To capture variation in environmental conditions during pre-natal and early post-natal development (early-life conditions), we took 3-year means of each environmental variable, centred on the birth year, a method previously used to show variation in reproductive performance in relation to crop yields around birth [23]. To investigate effects of prevailing conditions on mortality and reproduction, we used the environmental variables recorded in that year (current conditions). This allowed us to determine the immediate impact of environmental conditions on survival and reproduction.

### Statistical analysis

Initially, we used *E* and spring temperature as measures of environmental variation. We included data from individuals born from 1751 and tracked until 1877, excluding individuals who disappeared from our records, who emigrated during the study period, or who had unknown parental identity. Spring temperature data were available for all years, but data on infant mortality (*E*) were not due to damage of census records (Fig. 1A).

### Individual mortality

We analysed individual mortality in each year of life using binomial generalized linear mixed-effects models with logit link functions in the package lme4 [24] in R 2.13.0. Mortality was scored as 0 (survived to the end of the year) or 1 (died before the year ended). Data were split into three categories: children aged 1–15 years; adult males and adult females (aged >15 years). Children were analysed separately because their mortality rates differed from adults, and adults were divided by sex because mortality rates and life-history trajectories differed between the sexes. Analysing child mortality is important for testing the PAR hypothesis, since the fitness benefits of a PAR do not have to be expressed in adulthood: fitness benefits of altering development to maximize survival in early life may be favoured by selection even if a change in the environment is associated with late-life metabolic disease, because selection in early life is stronger than in later life [5]. We analysed adult mortality to test the hypothesis that PARs would maximize fitness across a long time span [3, 6]. We analysed mortality in 39 883 observation-years from 4251 children; 22 546 records from 1028 adult females and 22 304 records from 1008 adult males.

We initially ran models controlling for confounding variables, including study parish and social class as categorical fixed effects, with individuals classed by occupation as rich, middle-class or poor [25]. Twin status (singleton or twin) accounted for lower survival of twins [14], and birth order (firstborn or subsequent) inheritance bias [23]. We included linear and quadratic age in all models, divided by 100 to aid convergence in models of adults. For children, we included sex as a categorical fixed effect. We included random effects of individual identity, maternal identity and year to account for repeated measures and variation between individuals, families and years. We also repeated the analyses with birth year as a fixed effect and a random effect, but in all models, it was not significant and did not affect the results. We therefore present models excluding birth year. The full model was compared against a model where a variable of interest was absent. When dropping a variable increased the Akaike Information Criterion (AIC) value by >2, we regarded inclusion of the variable as improving the statistical fit of the model. Variables which, when dropped, resulted in an increase of <2 or a decrease in AIC value were not considered to improve model fit and were removed to attain a ‘base’ model for each data subset.

*E* and spring temperature were then added to the model separately; no model contained both. We examined independent effects of current and early-life conditions before adding their interaction. We added interactions with social class, and in analysis of children we tested interactions between environmental variables and age. Quadratic effects of environmental variables were tested but not supported. Models were compared as described above.

### Female annual reproductive success

Full evaluation of the fitness consequences of PARs also requires consideration of the other main fitness component, namely reproduction. To test the prediction that poor early conditions improve reproductive ability in poor later years relative to in favourable later years, we analysed female annual reproductive success (ARS). ARS was analysed as a binomial trait with a logit link function, where females either did (1) or did not (0) produce at least one child in a given year. We only included married women aged 16–45 years, since 97% of children were born during marriage and 99% between these ages [23]. We analysed 7695 records from 629 females born 1752–1856. Model selection proceeded as above.

Parameter estimates for all base models are shown in the Supplementary Information (Supplementary Table S1). In the Supplementary Tables and in the ‘Results’ section, parameter estimates ±1 standard error (SE) are given on the logit scale, as calculated by the mixed-effects models. In addition, we provide parameter estimates for key variables in all models in Supplementary Tables S4–S7.

## RESULTS

### Child mortality

**Figure 2.**
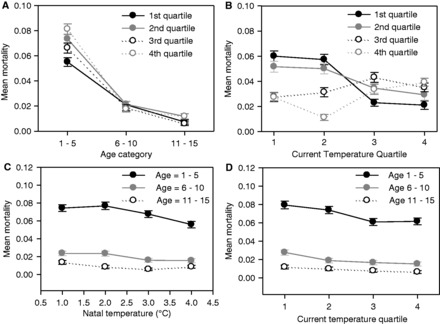
The effects of environmental conditions on child mortality were modified by age and early-life conditions. The plotted data shows interactions between variables which improved the fit of models of child mortality. (**A**) In older children, early-life *E* was not associated with mortality, while in younger children, higher early-life *E* (fourth quartile) was associated with higher mortality; (**B**) where current temperatures were warmer, mortality was independent of early-life temperature (ELT), but where current temperatures were cold, cooler early-life temperatures (first quartile) were associated with higher mortality; (**C**) mortality in older children was relatively unaffected by early-life temperature, but younger children showed higher mortality if they were born in cold years; (**D**) mortality in older children was slightly higher if current temperatures were lower, but in younger children, the effect of cooler temperatures on mortality was much stronger. Points show mean mortality, bars represent ± 1 SE.

Child mortality was higher in years of higher infant mortality (*E*) (Model 1 and Table 1; current *E* = 3.6997 ± 0.3662), and being born in high *E* years was associated with higher subsequent childhood mortality (Model 2 and Table 1; early-life *E* = 1.3827 ± 0.5734). Current *E* was a stronger determinant of childhood mortality since Model 3, containing both early-life and current *E*, was not an improvement on Model 1. The early-life *E* model was statistically improved by an interaction with age (Model 8), which suggested that only the youngest children experienced higher mortality when born in higher *E* years (Fig. 2A). The interaction between early-life and current *E* was not statistically supported (Model 4; estimate = 2.7897 ± 3.2015).

**Table 1.** Models investigating associations between environmental variation and child mortality

No.	Model	*E*		Spring temperature	
		AIC	ΔAIC	AIC	ΔAIC
0	BASE	11269.96	0.00	11269.96	0.00
1	BASE + current	**11171.60**	**−98.36**	11266.15	***−***3.81
2	BASE + early-life	11266.26	**−**3.70	11261.23	**−**8.73
3	BASE + early-life + current	11173.13	**−**96.83	11260.05	**−**9.91
4	BASE + early-life:current	11174.38	**−**95.58	11259.12	**−**10.84
5	BASE + social:current	11172.64	**−**97.32	11269.33	**−**0.63
6	BASE + social:early-life	11268.85	**−**1.11	11260.90	**−**9.06
7	BASE + age:current	11172.50	**−**97.46	11265.90	**−**4.06
8	BASE + age:early-life	11260.88	**−**9.08	11258.65	**−**11.31
9	BASE + age:(current + early-life)	–	–	**11254.75**	**−15.21**
10	BASE + current + age:early-life	11172.93	**−**97.03	–	–
11	BASE + age:(current + early-life) + Current:early-life	–	–	11255.17	**−**14.79

The table presents a comparison of binomial generalized linear mixed-effects models testing the effects of early-life and current environmental conditions and their interactions on mortality in children (aged 1–15 years). The numbered models, where current and early-life effects were either *E* (parish-wide infant mortality) or spring temperature, were compared with each other using AIC values. The best-supported models for each of *E* and spring temperature have the lowest AIC value and are shown in bold; the ΔAIC values are shown relative to the base model. ‘+’ indicates additional terms in the model structure, while ‘*X*:*X’* indicates an interaction term between *X* and *X*.

Current and early-life spring temperatures were both negatively associated with child mortality (Model 3 and Table 1). Mortality was higher in years with lower spring temperatures (current temperature = −0.0874 ± 0.0487), and in individuals experiencing lower early-life temperature (estimate = −0.1120 ± 0.0394). The early-life-by-current temperature interaction (Model 4) statistically improved model fit (estimate = 0.0472 ± 0.0277) and suggested that current spring temperature was not associated with mortality in individuals born in years with a warm spring, but individuals born during cooler years had higher mortality if the current year’s spring was cooler (Fig. 2B). The statistically best-supported model contained interactions between age and both early-life and current temperature (Model 9 and Table 1) and suggested that the increases in mortality with cooler early-life (interaction with age: estimate = −0.0168 ± 0.0094) and current (interaction with age: estimate = −0.0117 ± 0.0065) spring temperatures were stronger in younger individuals (Fig. 2C and D). These results do not meet the predictions of the PAR hypothesis, since they suggest that the highest mortality was experienced by individuals born in years of low spring temperature and experiencing low later-life spring temperature. Parameter estimates for all variables are shown in Supplementary Table S4.

### Adult mortality

**Figure 3.**
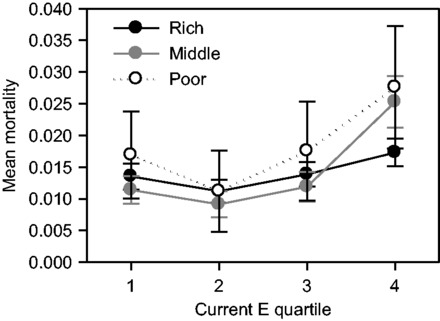
The effect of current *E* on adult female mortality was dependent on social class. The plotted data demonstrate that at low *E*, adult female mortality in the three socioeconomic classes are relatively similar; however, in years of high infant mortality, adult female mortality is higher in the poor and middle classes than in the rich class, which remains relatively unaffected. Points show mean mortality for each social class in the four quartiles of current *E* ±1 SE.

In adult females, current *E* was positively associated with mortality (Model 1 and Table 2), suggesting that years of high infant mortality coincided with high adult mortality (estimate = 3.0317 ± 0.6559). The statistically best-supported model contained a social class-by current *E* interaction (Model 5), suggesting that poor and middle-class females experienced a greater mortality increase with increasing *E* than did rich females (Fig. 3). The statistically best-supported spring temperature model (Model 2 and Table 2) suggested that mortality in adulthood was negatively associated with early-life spring temperature (estimate = −0.2648 ± 0.1065). We found no statistical support for the prediction that the lowest mortality would be found where early-life and current conditions matched (Model 4; early-life:current *E* estimate = −8.9703 ± 8.1053; early-life:current temperature estimate = 0.0733 ± 0.0584). Parameter estimates for all terms are shown in Supplementary Table S5.

**Table 2.** Models investigating associations between environmental variation and adult female mortality

No.	Model	*E*		Spring temperature	
		AIC	ΔAIC	AIC	ΔAIC
0	BASE	3106.81	0.00	3106.81	0.00
1	BASE + current	3089.80	−17.01	3108.69	1.88
2	BASE + early-life	3106.75	−0.06	**3101.62**	−**5.19**
3	BASE + early-life + current	3089.32	−17.49	−	−
4	BASE + early-life:current	3089.82	−16.98	3103.49	−3.32
5	BASE + social:current	**3087.07**	−**19.74**	3108.48	1.68
6	BASE + social:early-life	3107.89	1.09	3104.34	−2.46

The table shows a comparison of binomial generalized linear mixed-effects models testing the effects of early-life and current environmental conditions and their interactions on annual individual mortality in adult females aged >15 years. The numbered models, where current and early-life effects were either *E* or spring temperature, were compared with each other using AIC values. The best-supported models for each of *E* and spring temperature have the lowest AIC value and are shown in bold; the ΔAIC values are shown relative to the base model. ‘+’ indicates additional terms in the model structure, while ‘*X*:*X’* indicates an interaction term between *X* and *X*.

The statistically best-supported model for adult male mortality suggested a positive association with current *E* (estimate = 2.3511 ± 0.6686; Supplementary Table S2). Models with early-life or current temperature did not receive higher statistical support than the base model. We again found no statistical support for the prediction of maximal survival where early-life conditions matched current conditions (early-life:current *E* estimate = 6.3224 ± 5.8442; early-life:current temperature estimate = −0.0596 ± 0.0528). Parameter estimates for all terms are shown in Supplementary Table S6.

### Female reproductive success

Neither early-life nor current environmental conditions nor their interaction were associated with female ARS (Supplementary Table S3). This failed to support our prediction that fertility would be maximized where early- and later-life conditions matched. There was no evidence that the interaction between early-life and current *E* (estimate = 2.6833 ± 4.3104) or spring temperature (estimate = −0.0134 ± 0.0227) improved model fit. Parameter estimates for all terms are shown in Supplementary Table S7.

We repeated all analyses with environmental variables characterized as two-level factors, dividing them into years of below or above median value. This allowed us to explicitly test the predictions outlined in the ‘Introduction’ section. The results are presented in Supplementary Appendix S2. They were largely identical to results where environmental variables were characterized as continuous covariates and, importantly, not consistent with the prediction that survival and reproductive success would be maximized when environmental conditions matched between early and later life.

### Analysis with grain yields

Under the PAR hypothesis, nutrition is the crucial determinant of developmental trajectory. Both *E* and spring temperature are likely associated with nutrition [18, 26], but we analysed a more direct measure of food availability, by testing associations between crop yield data collected 1804–51 and childhood mortality in Ikaalinen. In summary, individual mortality was highest when both early-life and current crop yields were low. Once again, there was no support for the prediction that survival would be maximized when early-life and current conditions matched (Supplementary Appendix S3).

## CONCLUSIONS AND IMPLICATIONS

Several hypotheses have attempted to explain how environmental conditions experienced during development shape later-life health and fitness, and one of the most cited in recent years is the PAR hypothesis. This proposes that developmental plasticity is favoured by selection because it maximizes survival and fertility where conditions in later life are similar to those during development, with the negative effect of metabolic disease in non-matching conditions insufficient to exert negative selection on plasticity [6, 7].We used longitudinal data on individual mortality, reproduction and environmental variation to test this key prediction of the PAR hypothesis. This is, to our knowledge, the first empirical test in humans where associations with fitness over a range of environmental conditions during early development and later life have been investigated. We found that poorer early-life and current conditions were associated with higher mortality. Meanwhile, none of our analyses supported the prediction of the PAR hypothesis that survival and reproductive success would be higher in ‘matched’ early and later-life environmental conditions compared with where early- and later-life conditions matched.

### Associations between environmental variation and fitness

‘Current’ environmental conditions were associated with variation in mortality, but not female ARS. *E* was associated with mortality in both adults and children, suggesting that it represents environmental variation with fitness consequences for both. Therefore, it seems that our lack of support for the PAR hypothesis does not result from our environmental measures being a poor reflection of living conditions among the study subjects. Although the principle causes of mortality between children and adults differ to some extent [27, 28], *E* may be related to variation in both child and adult mortality because it was associated with variation in environmental conditions which affected both: for example, food shortage, or transmission of diseases affecting adults (e.g. tuberculosis), children (e.g. smallpox) or both (typhoid fever). Spring temperature was also associated with mortality in children and adults, suggesting that lower spring temperatures increased mortality risk, potentially due to lower rye yield, cold or disease. Finally, higher rye yields were associated with lower child mortality, particularly among the poorest social class (Supplementary Appendix S3). The finding that neither current *E* nor spring temperature was associated with female ARS makes it difficult to infer whether our failure to find a difference between matching and contrasting early-life and current environments refutes the mismatch hypothesis. If the probability of giving birth is not associated with the range of environmental variation measured here, then adaptive benefits of prediction are unlikely to be manifest in improved reproduction. However, a previous study found that crop yields around birth affected subsequent female probability of marriage and offspring survival [23]. Unfortunately, we were unable to assess the effects of crop yields on adult fitness measures due to the short time series of the available data (Supplementary Appendix S3). Nonetheless, the lack of effects on reproductive rate stresses the importance of significant effects on survival detected in this study when evaluating the overall fitness consequences of matching and mismatching early and later environments [29].

We also found evidence that early-life environmental conditions were associated with later mortality, although again there was no strong association with ARS. We captured pre- and post-natal environmental conditions by using a 3-year mean centred on the year of birth, since in mammals with extended parental care, events during gestation and post-parturition can affect later-life fitness [1]. Higher early-life *E* was associated with higher mortality in children, and lower early-life spring temperature was associated with higher mortality in children and adult females. In children, spring temperature effects were strongest at younger ages, potentially suggesting that effects of low early-life temperatures were short-lived. This observation is consistent with strong natural selection in early life in this population [29]. Alternatively, interactions between early-life conditions and age could suggest that the most environment-sensitive children die earlier. Finally, autocorrelation between environmental conditions measured in consecutive years may contribute to the observed associations. This is most likely to be true during the first year after birth, when environmental conditions are measured in consecutive years and the early-life effect could reflect current conditions as well as early-life conditions. However, for spring temperature, a model with both early-life and current conditions was a better fit than models with either variable alone, suggesting independent effects. In addition, the best model contained interactions between age and both early-life and current temperature, again suggesting independent effects of early-life and current conditions. In the second year of life, the correlation breaks down, and the fact that early-life temperature effects are seen on adult female mortality again suggests that autocorrelation does not explain all of the associations between early-life conditions and mortality in children.

The only instance of an interaction between early-life and current conditions was found for child mortality. Individuals experiencing lower spring temperature in later childhood showed higher mortality if they had experienced lower spring temperatures around birth than if they had experienced higher temperatures around birth (Fig. 2B and Supplementary Table S4). Based on the PAR hypothesis, we predicted the opposite. Surprisingly, Fig. 2B shows higher mortality under warmer later-life spring temperatures among individuals that experienced warm early-life temperatures than those that experienced low early-life temperatures. However, the difference is slight compared with the large difference in mortality at low later-life temperatures between individuals born under low spring temperatures versus those born under warmer conditions. Importantly, the observations under higher temperatures (Fig. 2B) are not consistent with the predictions of the PAR hypothesis, which would predict higher fitness under matched early-life and current conditions [8].

A further possibility is that the choice of study population may influence the results of this correlational study. The environmental conditions in which human life histories evolved may be different from those represented here: for instance, conditions in equatorial regions may have been more stable. Despite this, it is clear that variation in environmental conditions across years and seasons have been experienced in human populations across the world, extending back to pre-history [30]. It is interesting to speculate on the differing pressures exerted on populations by differing degrees of environmental variation, and whether these could change our assumptions about adaptive plasticity. For instance, in a very stochastic environment, we may expect weaker selection for development to match adulthood environmental conditions, because there would be a small probability of conditions matching. Meanwhile, in very stable environments, we may expect all individuals to show the same developmental trajectory, because developing along a different trajectory is selected against. Therefore, only where the environment fluctuates, but does so in a predictable manner, should we expect to see PARs favoured by selection.

### Alternatives to the PAR hypothesis

Observations of increased later-life disease risk in individuals who experience poor environmental conditions in early life and improved conditions in later life have increasingly been interpreted as consistent with the predictions of the PAR hypothesis [6]. These patterns have also been interpreted through the ‘silver spoon’ model, which proposes that adverse health and fitness outcomes are due to constrained development *in utero*, and that poor developmental conditions reduce ability to plastically adapt to adverse later-life conditions [31]. This is consistent with observations in wild animal populations, where poor early-life conditions are associated with reduced reproductive success and growth rates in adulthood [1]. One limitation of previous studies on humans has been that the effects of varying early conditions on later fitness have largely been tested in favourable later environments, perhaps leading to premature conclusions concerning the benefits of adaptive plasticity in poor adult environments. The ‘silver spoon’ model is a more parsimonious explanation for the link between adverse early conditions and poor later-life health than the PAR hypothesis. In order for prediction to maximize reproductive success, environmental conditions must be stable enough for information about the developmental environment to remain valid during reproductive life, as shown by theoretical work demonstrating that plasticity should only be favoured by selection where the association between early- and later-life conditions is strong [32]. This is most likely to be true for short-lived animals inhabiting stable environments, where such prediction only needs to be correct over a few months [33, 34]. It is less likely to be true in humans, where prediction of the future environment would have to be accurate over decades to provide fitness benefits at reproductive ages [35, 36]. Conditions must also vary sufficiently for prediction to confer a fitness benefit, since the costs of altering development and of making an incorrect prediction must be offset by correct prediction if prediction is to be favoured by selection [37]. However, we were unable to test whether prediction maximized survival of the foetus or during the critical first year of life [15]. Such ‘short-term’ prediction remains an attractive hypothesis but requires more empirical tests.

Finally, we acknowledge some limitations of the data that constrain our ability to definitely test the validity of the PAR hypothesis. Our annual measurements make it impossible to separate pre- from post-natal conditions or to determine trimester-specific conditions. The 3-year average was taken to capture environmental variation during pre- and post-natal development, but this method is unable to identify specific windows in which conditions may drive development irrespective of conditions at other stages of ontogeny [3]. We repeated the analysis using environmental conditions in the year of birth and this did not provide any support for the PAR hypothesis, but if a short period of development is crucial, this should not be surprising. Due to this limitation and the fact that the benefits of PARs may only be apparent in populations experiencing predictable variation, we cannot definitively disprove the PAR hypothesis, although the data and analysis we have conducted provide no evidence in favour of it. We hope that the evolutionary predictions of the hypothesis continue to be tested using a broad range of approaches and making use of a diverse array of available data, since only then we will increase our understanding of the selective pressures on developmental plasticity and be able to make inferences about potential effects on health.

We have used data from longitudinally monitored human populations to examine the effects of environmental conditions in early and later life on fitness in order to empirically test predictions of the PAR hypothesis. We did not find that poor early-life conditions maximized fitness under similar conditions in later life; instead, poor early-life conditions were associated with lower survival and reproduction in later life irrespective of later-life conditions. We have taken an evolutionary ecological approach to the study of the evolutionary origins of metabolic disease, investigating the role of early- and later-life environmental conditions in shaping human life history, and have found no evidence in support of the PAR hypothesis. We suggest that future studies focus on ‘short-term’ predictive responses, to test the hypothesis that the adverse health or fitness outcomes of poor nutrition have evolved through pleiotropic positive effects on early-life survival. Natural selection is strongest in early life, and prediction of post-natal environmental conditions based on gestational conditions is most likely to be accurate in the very short term [8, 36]. Therefore, it is during early life that predictive developmental plasticity is most likely to be adaptive and to be favoured by natural selection.

## SUPPLEMENTARY DATA

Supplementary data is available at *EMPH* online.

## FUNDING

European Research Council (R/120448-11-1) and a Royal Society University Research Fellowship (R/116946-11-1) (to V.L.).

**Conflict of interest**: None declared.

## Supplementary Material

Supplementary Data
