# Supplementary material for: Testing the evolutionary basis of the predictive adaptive response hypothesis in a preindustrial human population
Source: Evol Med Public Health. 2013 Apr 18;2013(1):106–17. doi: 10.1093/emph/eot007 (PMC3868390; doi:10.1093/emph/eot007)
Supplement: Supplementary Data [file supp_2013_1_106__index.html]

Testing the evolutionary basis of the Predictive Adaptive Response hypothesis in a preindustrial human population — Testing the evolutionary basis of the predictive adaptive response hypothesis in a preindustrial human population — Supplementary Data 

# Testing the evolutionary basis of the predictive adaptive response hypothesis in a preindustrial human population

## Supplementary Data

files

**Files in this Data Supplement:**

- Supplementary Data - docx file
