# Supplementary material for: Testing the evolutionary basis of the predictive adaptive response hypothesis in a preindustrial human population
Source: Evol Med Public Health. 2013 Apr 18;2013(1):106–17. doi: 10.1093/emph/eot007 (PMC3868390; doi:10.1093/emph/eot007)
Supplement: Supplementary Data [file supp_eot007_Hayward_Lummaa_PAR_SI.docx]

**Supplementary Information:** *Testing the Evolutionary Basis of the Predictive Adaptive Response Hypothesis in a Preindustrial Human Population*

**Appendix S1: Supplementary tables from the main text.**

*Tables containing results from the analysis described in the main text*

**Table S1:** **Parameter estimates for the base models*.***

The table shows parameter estimates for confounding variables we accounted for in the ‘base’ models for each analysis in the main text. In all mortality analyses, father’s social class is used; in analysis of female annual reproductive success (ARS), the social class of the focal female’s husband is used. Parameter estimates and standard errors (S.E.) shown are from the mixed-effects models on the logit scale.

|  | Child mortality | |  | Adult female mortality | |  | Adult male mortality | |  | Female ARS | |  |
| --- | --- | --- | --- | --- | --- | --- | --- | --- | --- | --- | --- | --- |
| **Variables** | **Estimate** | **S.E.** |  | **Estimate** | **S.E.** |  | **Estimate** | **S.E.** |  | **Estimate** | **S.E.** |  |
| *Fixed effects* |  |  |  |  |  |  |  |  |  |  |  |  |
| Intercept | -2.0277 | 0.1248 |  | -6.3172 | 0.5056 |  | -8.6464 | 0.3161 |  | -2.9649 | 0.6185 |  |
| Parish (Hiittinen) | 0.0000 | 0.0000 |  | 0.0000 | 0.0000 |  | 0.0000 | 0.0000 |  | 0.0000 | 0.0000 |  |
| Parish (Ikaalinen) | 0.3637 | 0.0917 |  | -0.7409 | 0.2931 |  | -0.6141 | 0.2734 |  | 0.2450 | 0.0854 |  |
| Parish (Kustavi) | -0.2877 | 0.1057 |  | -0.1401 | 0.2836 |  | 0.5007 | 0.2763 |  | -0.0503 | 0.0988 |  |
| Parish (Rymättlyä) | 0.0545 | 0.1069 |  | 0.4542 | 0.2814 |  | 0.6958 | 0.2616 |  | -0.2210 | 0.0974 |  |
| Social class (Rich) | 0.0000 | 0.0000 |  | 0.0000 | 0.0000 |  | 0.0000 | 0.0000 |  | 0.0000 | 0.0000 |  |
| Social class (Middle) | 0.1149 | 0.0663 |  | 0.2963 | 0.2246 |  | -0.1566 | 0.2141 |  | -0.0184 | 0.0688 |  |
| Social class (Poor) | 0.4528 | 0.1076 |  | 0.5455 | 0.4070 |  | 0.2623 | 0.3667 |  | -0.2049 | 0.1116 |  |
| Sex (Male) | ns | ns |  | ns | ns |  | ns | ns |  | ns | ns |  |
| Sex (Female) | ns | ns |  | ns | ns |  | ns | ns |  | ns | ns |  |
| Twin status (Single) | 0.0000 | 0.0000 |  | ns | ns |  | ns | ns |  | ns | ns |  |
| Birth status (Twin) | 0.6880 | 0.1136 |  | ns | ns |  | ns | ns |  | ns | ns |  |
| Birth order (0) | 0.0000 | 0.0000 |  | ns | ns |  | ns | ns |  | ns | ns |  |
| Birth order (1) | 0.1257 | 0.0704 |  | ns | ns |  | ns | ns |  | ns | ns |  |
| Age | -0.4689 | 0.0272 |  | -0.5499 | 2.2314 |  | 10.1407 | 0.5401 |  | 19.4322 | 3.9422 |  |
| Age² | 0.0179 | 0.0020 |  | 10.1407 | 2.4263 |  | ns | ns |  | -40.1868 | 6.1435 |  |
| *Random effects* |  |  |  |  |  |  |  |  |  |  |  |  |
| Individual identity | ns | ns |  | 2.8902 | 0.0530 |  | 2.2404 | 0.0466 |  | 0.1523 | 0.0156 |  |
| Maternal identity | 0.1761 | 0.0109 |  | ns | ns |  | ns | ns |  | ns | ns |  |
| Year | 0.3681 | 0.0540 |  | 0.0674 | 0.0250 |  | 0.1283 | 0.0341 |  | ns | ns |  |

**Table S2:** **Models investigating associations between environmental variation and adult male mortality.**

The table shows a comparison of binomial generalised linear mixed-effects models (GLMMs) of mortality for adult males aged 16 and over. Each of the numbered models was assessed where current and early-life effects were either E or spring temperature; models of E or spring temperature were compared to each other using AIC values. The best-supported model for each of E and spring temperature has the lowest AIC value and are shown *in* ***bold italics***; the ΔAIC values are shown relative to the base model. “+” indicates additional fixed effects, while “:” indicates an interaction between two fixed effects, and it is implied that the main effects are also included in the model.

|  |  | **E** | |  | **Spring temperature** | |
| --- | --- | --- | --- | --- | --- | --- |
| **No.** | **Model** | **AIC** | **ΔAIC** |  | **AIC** | **ΔAIC** |
| 0 | BASE | 3194.69 | 0.00 |  | ***3194.69*** | ***0.00*** |
| 1 | BASE + Current | ***3185.44*** | ***-9.25*** |  | 3196.09 | 1.40 |
| 2 | BASE + Early-life | 3196.06 | 1.37 |  | 3196.26 | 1.58 |
| 3 | BASE + Early-life:Current | 3187.99 | -6.69 |  | 3198.17 | 3.49 |
| 4 | BASE + Social:Current | 3188.70 | -5.98 |  | 3199.51 | 4.82 |
| 5 | BASE + Social:Early-life | 3200.32 | 5.64 |  | 3198.25 | 3.57 |

**Table S3:** **Models investigating associations between environmental variation and adult female annual reproductive success.**

The table shows a comparison of binomial generalised linear mixed-effects models of annual fecundity of females aged 16–45. Each of the numbered models was assessed where current and early-life effects were either E or spring temperature; models of E or spring temperature were compared to each other using AIC values. The best-supported model for each of E and spring temperature has the lowest AIC value and are shown *in* ***bold italics***; the ΔAIC values are shown relative to the base model. “+” indicates additional fixed effects, while “:” indicates an interaction between two fixed effects, and it is implied that the main effects are also included in the model.

|  |  | **E** | |  | **Spring temperature** | |
| --- | --- | --- | --- | --- | --- | --- |
| **No.** | **Model** | **AIC** | **ΔAIC** |  | **AIC** | **ΔAIC** |
| 0 | BASE | ***8577.60*** | ***0.00*** |  | ***8577.60*** | ***0.00*** |
| 1 | BASE + Current | 8578.82 | 1.22 |  | 8578.98 | 1.38 |
| 2 | BASE + Early-life | 8579.36 | 1.76 |  | 8579.58 | 1.98 |
| 3 | BASE + Early-life:Current | 8582.23 | 4.63 |  | 8582.63 | 5.02 |
| 4 | BASE + Social:Current | 8578.79 | 1.18 |  | 8582.57 | 4.97 |
| 5 | BASE + Social:Early-life | 8582.42 | 4.82 |  | 8581.28 | 3.67 |

| **Model** | **Intercept** | **Age** | **Age²** | **Current** | **Early-life** | **Current:Early-life** | **Current:Age** | **Early-life:Age** | **AIC** |
| --- | --- | --- | --- | --- | --- | --- | --- | --- | --- |
| 0 | -2.03(0.12) | -0.47(0.03) | 0.02(0.00) | - | - | - | - | - | 11269.96 |
| *a) Models of infant mortality (E)* | | |  |  |  |  |  |  |  |
| 1 | -2.92(0.15) | -0.4684(0.03) | 0.02(0.00) | 3.70(0.37) | - | - | - | - | 11171.60 |
| 2 | -2.35(0.19) | -0.4666(0.03) | 0.02(0.00) | - | 1.38(0.57) | - | - | - | 11266.26 |
| 3 | -3.01(0.20) | -0.4673(0.03) | 0.02(0.00) | 3.66(0.37) | 0.41(0.59) | - | - | - | 11173.13 |
| 4 | -2.87(0.26) | -0.4645(0.03) | 0.02(0.00) | 3.07(0.77) | -0.34(1.05) | 2.79(3.20) | - | - | 11174.38 |
| 7 | -2.86(0.16) | -0.4857(0.03) | 0.02(0.00) | 3.41(0.46) | - | - | 0.08(0.08) | - | 11172.50 |
| 8 | -2.63(0.21) | -0.3965(0.04) | 0.02(0.00) | - | 2.81(0.77) | - | - | -0.37(0.14) | 11260.88 |
| 9 | - | - | - | - | - | - | - | - | - |
| *b) Models of spring temperature* | | |  |  |  |  |  |  |  |
| 1 | -1.71(0.18) | -0.4693(0.03) | 0.02(0.00) | -0.12(0.05) | - | - | - | - | 11266.15 |
| 2 | -1.68(0.16) | -0.4662(0.03) | 0.02(0.00) | - | -0.13(0.04) | - | - | - | 11261.23 |
| 3 | -1.49(0.19) | -0.4665(0.03) | 0.02(0.00) | -0.09(0.05) | -0.11(0.04) | - | - | - | 11260.05 |
| 4 | -1.18(0.26) | -0.4562(0.03) | 0.02(0.00) | -0.22(0.09) | -0.24(0.08) | 0.05(0.03) | - | - | 11259.12 |
| 7 | -1.80(0.19) | -0.4460(0.03) | 0.02(0.00) | -0.08(0.05) | - | - | -0.01(0.01) | - | 11265.90 |
| 8 | -1.92(0.20) | -0.4193(0.04) | 0.02(0.00) | - | -0.04(0.06) | - | - | -0.02(0.01) | 11258.65 |
| 9 | -1.73(0.22) | -0.3946(0.04) | 0.02(0.00) | -0.05(0.06) | -0.05(0.06) | - | -0.01(0.01) | -0.02(0.01) | 11254.75 |

**Table S4: Parameter estimates for all models of child mortality**

The table shows parameter estimates from models of child mortality, a comparison of which is shown in Table 1 in the main text; the model numbers refer to Table 1. Interactions with social class are not shown because they were never significant and due to restrictions in the dimensions of the table. For each model, the estimates from the mixed-effect model are shown on the original logit scale, with the estimated standard error in parentheses.

**Table S5: Parameter estimates for all models of adult female mortality**

The table shows parameter estimates from models of adult female mortality, a comparison of which is shown in Table 2 in the main text; the model numbers refer to Table 2. For each model, the estimates from the mixed-effect model are shown on the original logit scale, with the estimated standard error in parentheses. Estimates and standard errors are shown to only 2 decimal places for economy of space.

| **M** | **Int.** | **Social (M)** | **Social (P)** | **Current** | **Early-life** | **Cur:EL** | **Current:M** | **Current:P** | **Early-life:M** | **Early-life:P** | **AIC** |
| --- | --- | --- | --- | --- | --- | --- | --- | --- | --- | --- | --- |
| 0 | -6.32(0.51) | 0.30(0.22) | 0.55(0.41) | - | - | - | - | - | - | - | 3106.8 |
| *a) Models of infant mortality (E)* | | | | |  |  |  |  |  |  |  |
| 1 | -6.89(0.52) | 0.31(0.23) | 0.54(0.41) | 3.03(0.66) | - | - | - | - | - | - | 3089.8 |
| 2 | -5.86(0.61) | 0.29(0.22) | 0.56(0.41) | - | -2.18(1.57) | - | - | - | - | - | 3106.8 |
| 3 | -6.40(0.62) | 0.30(0.23) | 0.56(0.41) | 3.07(0.66) | -2.39(1.58) | - | - | - | - | - | 3089.3 |
| 4 | -6.77(0.71) | 0.30(0.23) | 0.55(0.41) | 4.92(1.77) | -0.62(2.23) | -8.97(8.11) | - | - | - | - | 3089.8 |
| 5 | -6.75(0.54) | -0.32(0.35) | 0.48(0.64) | 2.03(0.85) | - | - | 3.39(1.41) | 0.33(2.53) | - | - | 3087.1 |
| 6 | -6.06(0.63) | 1.10(0.60) | 0.11(1.07) | - | -1.31(1.78) | - | - | - | -4.63(3.18) | 2.39(4.94) | 3107.9 |
| *b) Models of spring temperature* | | | | |  |  |  |  |  |  |  |
| 1 | -6.26(0.52) | 0.29(0.22) | 0.54(0.41) | -0.02(0.06) | - | - | - | - | - | - | 3108.7 |
| 2 | -5.69(0.57) | 0.29(0.23) | 0.47(0.41) | - | -0.26(0.11) | - | - | - | - | - | 3101.6 |
| 3 | - | - | - | - | - | - | - | - | - | - | - |
| 4 | -5.15(0.70) | 0.29(0.23) | 0.46(0.41) | -0.23(0.18) | -0.41(0.16) | 0.07(0.06) | - | - | - | - | 3103.5 |
| 5 | -6.34(0.53) | 0.62(0.32) | 0.14(0.62) | 0.02(0.07) | - | - | -0.16(0.11) | 0.17(0.19) | - | - | 3108.5 |
| 6 | -5.66(0.64) | 0.08(0.66) | 1.56(1.27) | - | -0.28(0.14) | - | - | - | 0.08(0.22) | -0.43(0.47) | 3104.3 |

Abbreviations: M = model; Int. = model intercept; Social(M) = middle class; Social(P) = poor class; Cur:EL = interaction between current and early-life environment; Current:M = interaction between current environment and middle class; Current:P = interaction between current environment and middle class; Early-life:M = interaction between early-life environment and middle class; Early-life:P = interaction between early-life environment and poor class.

**Table S6: Parameter estimates for all models of adult male mortality**

The table shows parameter estimates from models of adult male mortality, a comparison of which is shown in Table S2 above; the model numbers refer to Table S2. For each model, the estimates from the mixed-effect model are shown on the original logit scale, with the estimated standard error in parentheses. Estimates and standard errors are shown to only 2 decimal places for economy of space.

| **M** | **Int.** | **Social (M)** | **Social (P)** | **Current** | **Early-life** | **Cur:EL** | **Current:M** | **Current:P** | **EL:M** | **EL:P** | **AIC** |
| --- | --- | --- | --- | --- | --- | --- | --- | --- | --- | --- | --- |
| 0 | -8.65(0.32) | -0.16(0.21) | 0.26(0.37) |  |  |  |  |  |  |  | 3194.7 |
| *a) Models of infant mortality (E)* | | | | |  |  |  |  |  |  |  |
| 1 | -9.23(0.36) | -0.15(0.22) | 0.27(0.38) | 2.35(0.67) | - | - | - | - | - | - | 3185.6 |
| 2 | -8.53(0.47) | -0.16(0.21) | 0.26(0.37) | - | -0.54(1.46) | - | - | - | - | - | 3196.1 |
| 3 | -8.77(0.58) | -0.16(0.22) | 0.27(0.38) | 0.92(1.53) | -2.07(1.97) | 6.32(5.84) | - | - | - | - | 3188 |
| 4 | -9.16(0.37) | -0.37(0.35) | 0.21(0.58) | 2.07(0.77) | - | - | 1.19(1.46) | 0.30(2.50) | - | - | 3188.7 |
| 5 | -8.57(0.49) | -0.06(0.53) | 0.67(0.99) | - | -0.33(1.61) | - | - | - | -0.52(2.75) | -2.44(5.53) | 3200.3 |
| *b) Models of spring temperature* | | | | |  |  |  |  |  |  |  |
| 1 | -8.50(0.35) | -0.16(0.21) | 0.26(0.36) | -0.04(0.06) | - | - | - | - | - | - | 3196.1 |
| 2 | -8.50(0.42) | -0.15(0.22) | 0.29(0.37) | - | -0.06(0.10) | - | - | - | - | - | 3196.3 |
| 3 | -8.77(0.56) | -0.16(0.21) | 0.24(0.37) | 0.14(0.17) | 0.07(0.15) | -0.06(0.05) | - | - | - | - | 3198.2 |
| 4 | -8.57(0.36) | -0.03(0.32) | 0.48(0.53) | -0.02(0.07) | - | - | -0.06(0.11) | -0.11(0.19) | - | - | 3199.5 |
| 5 | -8.82(0.50) | 0.54(0.64) | 1.20(1.07) | - | 0.05(0.13) | - | - | - | -0.24(0.21) | -0.35(0.38) | 3198.3 |

Abbreviations: M = model; Int. = model intercept; Social(M) = middle class; Social(P) = poor class; Cur:EL = interaction between current and early-life environment; Current:M = interaction between current environment and middle class; Current:P = interaction between current environment and middle class; EL:M = interaction between early-life environment and middle class; EL:P = interaction between early-life environment and poor class.

**Table S7: Parameter estimates for all models of adult female annual reproductive success**

This table shows parameter estimates from models of adult female annual reproductive success, a comparison of which is shown in Table S3 above; the model numbers refer to Table S3. For each model, the estimates from the mixed-effect model are shown on the original logit scale, with the estimated standard error in parentheses. Estimates and standard errors are shown to only 2 decimal places for economy of space.

| **M** | **Int.** | **Social (M)** | **Social (P)** | **Current** | **Early-life** | **Cur:EL** | **Current:M** | **Current:P** | **El:M** | **EL:P** | **AIC** |
| --- | --- | --- | --- | --- | --- | --- | --- | --- | --- | --- | --- |
| 0 | -2.96(0.62) | -0.02(0.07) | -0.20(0.11) | - | - | - | - | - | - | - | ***8578*** |
| a) Models of infant mortality (E) | | | | |  |  |  |  |  |  |  |
| 1 | -2.91(0.62) | -0.02(0.07) | -0.20(0.11) | -0.30(0.34) | - | - | - | - | - | - | 8578.8 |
| 2 | -2.91(0.63) | -0.02(0.07) | -0.20(0.11) | - | -0.27(0.55) | - | - | - | - | - | 8579.4 |
| 3 | -2.75(0.65) | -0.02(0.07) | -0.20(0.11) | -0.85(0.96) | -0.72(0.94) | 2.68(4.31) | - | - | - | - | 8582.2 |
| 4 | -3.01(0.62) | 0.14(0.14) | 0.13(0.22) | 0.24(0.46) | - | - | -0.94(0.72) | -2.08(1.18) | - | - | 8578.8 |
| 5 | -2.94(0.64) | -0.02(0.20) | 0.07(0.32) | - | -0.15(0.76) | - | - | - | -0.01(0.99) | -1.67(1.79) | 8582.4 |
| b) Models of spring temperature | | | | |  |  |  |  |  |  |  |
| 1 | -2.93(0.62) | -0.02(0.07) | -0.20(0.11) | -0.02(0.02) | - | - | - | - | - | - | 8579 |
| 2 | -2.95(0.63) | -0.02(0.07) | -0.20(0.11) | - | -0.00(0.03) | - | - | - | - | - | 8579.6 |
| 3 | -3.03(0.66) | -0.02(0.07) | -0.20(0.11) | 0.02(0.07) | 0.03(0.06) | -0.01(0.02) | - | - | - | - | 8582.6 |
| 4 | -2.96(0.62) | 0.03(0.12) | -0.11(0.20) | -0.00(0.03) | - | - | -0.02(0.04) | -0.04(0.07) | - | - | 8582.6 |
| 5 | -2.97(0.64) | 0.12(0.22) | -0.53(0.33) | - | 0.00(0.05) | - | - | - | -0.05(0.07) | 0.11(0.11) | 8581.3 |

Abbreviations: M = model; Int. = model intercept; Social(M) = middle class; Social(P) = poor class; Cur:EL = interaction between current and early-life environment; Current:M = interaction between current environment and middle class; Current:P = interaction between current environment and middle class; EL:M = interaction between early-life environment and middle class; EL:P = interaction between early-life environment and poor class.

**Appendix S2: Environmental variables as two-level factors**

*Analysis of mortality and reproduction with environmental variation characterised as high versus low*

**Supplementary analysis**

The models described in the main text characterise environmental variables across continuous scales, enabling us to consider the effects of the full range of environmental heterogeneity on mortality and reproduction. However, any detected associations could be due to the influence of years of extreme environmental conditions such as epidemics or famines. These effects are important determinants of patterns of selection, but may entirely account for the observed patterns beyond the usual range of environmental variation. Therefore, in order to ensure the effects we observed were not due to the extremes of environmental conditions, we repeated all analyses, characterising each of the environmental variables as a two-level factor, where “low” values were considered to be conditions at and below the median for each variable, and “high” was considered to be above the median of each variable. All base models were identical to those described in the main text and model selection proceeded in an identical fashion. These models also offer a more intuitive summary of how the interactions between “good” and “poor” conditions in early and later life influence survival and reproduction.

**Supplementary results**

*Child mortality*

The statistically best-supported model investigating the effects of annual infant mortality (E) contained interactions between age and both early-life and current E (model 9, Table S8). The interaction between age and early-life E was statistically supported in the analysis in the main text, but the interaction between age and current E was not, and suggested that the association between current E and mortality was strongest in the youngest individuals (Figure S1).

The models with spring temperature as a categorical fixed effect were in agreement with those where it was analysed as a continuous covariate. The statistically best-supported model contained interactions between age and both early-life and current temperature, as well as an interaction between early-life and current temperature (model 10, Table S8) that received marginal statistical support when spring temperature was analysed as a continuous covariate. The interaction suggested that individuals born in years of low spring temperature experienced lower mortality when the current temperature was high as opposed to when it was low, while no such effect was observed in individuals born in years of high spring temperature (Figure S2).

*Adult female mortality*

As with the models investigating associations between adult female mortality and spring temperature as a continuous covariate, the statistically best-supported model for adult female mortality with spring temperature as a two-level factor contained a main effect of early-life temperature (model 2; Table S9), suggesting that female mortality was highest in those born during years of low spring temperature (Early-life temperature (High) = -0.4506 ± 0.2054). The main effect of current E statistically improved model fit relative to the base model (model 1), suggesting that years of high infant mortality were also years of high adult mortality (Current E (High) = 0.5249 ± 0.1430). Unlike the models in the main text however, the interaction between social class and current E was not statistically supported; however, the interaction between early-life E and current E did receive marginal statistical support (model 3).

*Adult male mortality*

The statistically best-supported E model contained a main effect of current E (model 1; Table S10), suggesting that adult males experienced increased mortality when infant mortality was high (current E (High) = 0.3648 ± 0.1395). A model which also contained the effect of early-life E (model 3) was not statistically an improvement on the more parsimonious model 1. None of the models containing main effects or interactions involving spring temperature statistically improved the base model for adult male mortality.

*Female reproductive success*

None of the environmental variables statistically improved model fit; in this regard, the results were very similar to those treating environmental variables as continuous covariates.

*Ikaalinen child mortality*

The results of analysis of Ikaalinen child mortality, which included effects of E, spring temperature and crop yields (see Section S3), are shown in Table S11. The statistically best-supported model including effects of E contained the interaction between current E and social class, although this was only marginal. The interaction between early-life and current E, seen in the analysis in Section S3 did however not statistically improve model fit. As with the analysis in Section S3, there was evidence that both early-life and current temperatures were associated with mortality, and the best model contained both of these effects.

Unlike the analysis in Section S3, the models including rye grain yield as a categorical variable did not statistically improve model fit; however, the statistically best-supported barley grain model included the interaction between age and early-life barley which was seen in the continuous models in Section S3, although it was a stronger effect when fitted as a categorical variable. Finally, models containing mean crop yield were not statistically supported. Overall, the effects were weaker when these variables were fitted as categorical factors, suggesting that the effects may have been driven by particularly adverse years. In particular, the interactions in Figures S3 & S4 of Section S3 suggest that interactions were probably driven by the very poorest conditions.

**Table S8: Models investigating associations between environmental variation characterised as a categorical variable and child mortality**

The table shows a comparison of binomial generalised linear mixed-effects models of mortality for pre-reproductive individuals (aged 1–15). Each of the numbered models was assessed where current and early-life effects were either E or spring temperature; models of E or spring temperature were compared to each other using AIC values. The best-supported models for each of E and spring temperature have the lowest AIC value and are shown *in* ***bold italics***; the ΔAIC values are shown relative to the base model.

|  |  | **E** | |  | **Spring temperature** | |
| --- | --- | --- | --- | --- | --- | --- |
| **No.** | **Model** | **AIC** | **ΔAIC** |  | **AIC** | **ΔAIC** |
| 0 | BASE | 11269.96 | 0.00 |  | 11269.96 | 0.00 |
| 1 | BASE + Current | 11199.09 | -70.87 |  | 11268.36 | -1.60 |
| 2 | BASE + Early-life | 11271.89 | 1.93 |  | 11263.14 | -6.82 |
| 3 | BASE + Early-life + Current | - | - |  | 11262.96 | -7.00 |
| 4 | BASE + Early-life:Current | 11201.44 | -68.52 |  | 11260.04 | -9.92 |
| 5 | BASE + Social:Current | 11201.22 | -68.74 |  | 11271.89 | 1.93 |
| 6 | BASE + Social:Early-life | 11275.74 | 5.78 |  | 11262.63 | -7.33 |
| 7 | BASE + Age:Current | 11192.57 | -77.39 |  | 11269.80 | -0.16 |
| 8 | BASE + Age:Early-life | 11266.30 | -3.66 |  | 11261.70 | -8.26 |
| 9 | BASE + Age:(Current + Early-life) | **11192.12** | **-77.84** |  | 11261.82 | -8.14 |
| 10 | BASE + Age:(Current + Early-life) + Current:Early-life | - | - |  | **11259.36** | **-10.60** |

**Table S9:** **Models investigating associations between environmental variation as a categorical variable and adult female mortality.**

The table presents a comparison of binomial generalised linear mixed-effects models of adult female (aged 16+) mortality. Each model was assessed where current and early-life effects were either E or spring temperature; models of E or spring temperature were compared using AIC values. The best-supported models for each of E and spring temperature have the lowest AIC value and are shown *in* ***bold italics***; the ΔAIC values are shown relative to the base model.

|  |  | **E** | |  | **Spring temperature** | |
| --- | --- | --- | --- | --- | --- | --- |
| **No.** | **Model** | **AIC** | **ΔAIC** |  | **AIC** | **ΔAIC** |
| 0 | BASE | 3106.81 | 0.00 |  | 3106.81 | 0.00 |
| 1 | BASE + Current | 3093.35 | -13.45 |  | 3108.80 | 1.99 |
| 2 | BASE + Early-life | 3107.75 | 0.94 |  | ***3103.44*** | ***-3.37*** |
| 3 | BASE + Early-life:Current | ***3092.15*** | ***-14.66*** |  | 3106.64 | -0.17 |
| 4 | BASE + Social:Current | 3095.76 | -11.05 |  | 3109.20 | 2.39 |
| 5 | BASE + Social:Early-life | 3108.00 | 1.19 |  | 3106.54 | -0.26 |

**Table S10: Models investigating associations between environmental variation as a categorical variable and adult male mortality.**

The table compares binomial generalised linear mixed-effects models of adult male (aged 16+) mortality. Each model was assessed where current and early-life effects were either E or spring temperature; models of E or spring temperature were compared using AIC values. The best-supported models for each of E and spring temperature have the lowest AIC value and are shown *in* ***bold italics***; the ΔAIC values are shown relative to the base model.

|  |  | **E** | |  | **Spring temperature** | |
| --- | --- | --- | --- | --- | --- | --- |
| **No.** | **Model** | **AIC** | **ΔAIC** |  | **AIC** | **ΔAIC** |
| 0 | BASE | 3194.69 | 0.00 |  | ***3194.69*** | ***0.00*** |
| 1 | BASE + Current | 3189.34 | -5.34 |  | 3196.58 | 1.90 |
| 2 | BASE + Early-life | 3194.32 | -0.37 |  | 3195.92 | 1.24 |
| 3 | BASE + Early-life + Current | ***3188.77*** | ***-5.92*** |  | - | - |
| 4 | BASE + Early-life:Current | 3189.79 | -4.90 |  | 3198.34 | 3.66 |
| 5 | BASE + Social:Current | 3192.17 | -2.52 |  | 3200.03 | 5.34 |
| 6 | BASE + Social:Early-life | 3196.90 | 2.22 |  | 3199.21 | 4.52 |

**Table S11:** **Models investigating associations between environmental variation as a categorical variable and Ikaalinen child mortality.**

The table presents a comparison of binomial generalised linear mixed-effects models of mortality for Ikaalinen children (aged 1–15), where environmental variables are characterised as two-level categorical factors. Each of the numbered models was assessed where current and early-life effects were E, spring temperature, rye yield, barley yield or mean crop yield, and models of the different environmental variables were compared to each other separately using AIC values. The best-supported models for each of E and spring temperature have the lowest AIC value and are shown *in* ***bold italics***; the ΔAIC values are shown relative to the base model.

|  |  | **E** | |  | **Spring temperature** | |  | **Rye** | |  | **Barley** | |  | **Mean Crop** | |
| --- | --- | --- | --- | --- | --- | --- | --- | --- | --- | --- | --- | --- | --- | --- | --- |
| **No.** | **Model** | **AIC** | **ΔAIC** |  | **AIC** | **ΔAIC** |  | **AIC** | **ΔAIC** |  | **AIC** | **ΔAIC** |  | **AIC** | **ΔAIC** |
| 0 | BASE | 2760.45 | 0.00 |  | 2760.45 | 0.00 |  | ***2760.45*** | ***0.00*** |  | 2760.45 | 0.00 |  | 2760.45 | 0.00 |
| 1 | BASE + Current | 2760.74 | 0.29 |  | 2758.13 | -2.32 |  | 2761.80 | 1.35 |  | 2762.40 | 1.95 |  | 2762.07 | 1.62 |
| 2 | BASE + Early-life | 2762.44 | 1.99 |  | 2754.59 | -5.86 |  | 2761.66 | 1.21 |  | 2759.98 | -0.47 |  | ***2760.06*** | ***-0.39*** |
| 3 | BASE + Early-life + Current | 2762.69 | 2.24 |  | ***2753.79*** | ***-6.66*** |  | 2763.18 | 2.73 |  | 2761.76 | 1.31 |  | 2761.90 | 1.45 |
| 4 | BASE + Social:Current | ***2759.35*** | ***-1.10*** |  | 2761.54 | 1.09 |  | 2762.44 | 1.99 |  | 2759.31 | -1.14 |  | 2762.07 | 1.62 |
| 5 | BASE + Social:Early-life | 2766.18 | 5.73 |  | 2757.43 | -3.02 |  | 2762.59 | 2.14 |  | 2758.70 | -1.75 |  | 2763.78 | 3.33 |
| 6 | BASE + Age:Current | 2761.31 | 0.86 |  | 2759.73 | -0.72 |  | 2763.18 | 2.73 |  | 2764.40 | 3.95 |  | 2763.65 | 3.20 |
| 7 | BASE + Age:Early-life | 2764.40 | 3.95 |  | 2755.18 | -5.27 |  | 2763.62 | 3.17 |  | ***2757.94*** | ***-2.51*** |  | 2761.87 | 1.42 |
| 8 | BASE + Early-life:Current | 2764.63 | 4.18 |  | 2757.12 | -3.33 |  | 2763.86 | 3.41 |  | 2758.73 | -1.72 |  | 2763.44 | 2.99 |

**Figure S1:** **High early-life E was associated with higher child mortality, especially amongst the youngest individuals.**

The figure shows plotted data showing that mortality in children was lower where current E (infant mortality) was Low than when it was High. This effect was particularly strong in younger children. Note that in analysis, age was treated as a continuous quadratic function; age groups were binned for ease of interpretation of plots.

**Figure S2: Child mortality was highest amongst individuals experiencing a combination of low early-life and current temperatures.**

The plotted data shows that child mortality was highest when individuals experienced both low early-life and current temperatures; the strongest effect of early-life temperature on child mortality were found in individuals that experienced low early-life temperature. Bars show mean mortality as a function of early-life and current temperature ± 1S.E.

**Appendix S3: Supplementary analysis using grain yields**

*Supplementary analysis of Ikaalinen child mortality using crop grain yields as a measure of environmental variation*

**Supplementary analysis**

The key to the predictive adaptive response hypothesis (PAR) is that nutrition is the major determinant of developmental trajectory. In our data, E (infant mortality) and spring temperature are associated with nutrition, since E captures variation in all-cause mortality, and spring temperature is positively associated with rye yield. However, E is associated with causes of death unrelated to nutrition, such as epidemic disease, the biggest killer in preindustrial Europe [[1-3](#_ENREF_1)], and spring temperature will potentially be associated with cold stress and disease prevalence. Therefore, in addition to these measures of environmental conditions, we wished to include a measure of food availability in our analyses. We used grain figures (the amount of grain harvested as a multiple of the amount sown) of rye and barley, the two most important crops in Finnish agriculture, which provided up to 80% of the energetic requirements of working people [[4](#_ENREF_4)]. These had to be of a sufficient time series to examine interactions between early and later life, and had to be located within 50km of the study areas to provide the most accurate measure of local conditions [[5](#_ENREF_5)]. The only data fulfilling these criteria were the grain figures from the estate of Valkila, which is located less than 50km from the centre of Ikaalinen [[6](#_ENREF_6)] and is available for the period 1805-1875. These data are associated with food availability, since low yields are associated with famine [[7](#_ENREF_7)], and previous studies in this population has shown that low grain yields are associated with both mortality and reproduction [[8](#_ENREF_8), [9](#_ENREF_9)]. We repeated the analysis of child mortality on a subset of the data used in the main text for Ikaalinen children only, ensuring we captured as much of the variation in conditions and as many individuals as possible; analysing adults as well would have severely reduced the number of individuals due to the short time frame which is considerably lower than the maximum lifespan. As noted in the main text, E data were available only for Ikaalinen up to 1851, so we repeated all analysis of child mortality on 9272 records from 1091 individuals aged 1-15, born to 432 mothers over 41 years. We repeated analysis using spring temperature and E, and also three crop variables: rye yield, barley yield, and the mean of the two. Models were selected by comparing AIC values and selecting the models with lower AIC values as in the main text.

**Supplementary results**

The models assessing child mortality in Ikaalinen are shown in Table S12. The statistically best-supported model containing effects of E contained an interaction between early-life and current E, which suggested that individuals had the highest mortality if they experienced high early-life E and were currently in a high E year (Figure S3). The models of spring temperature produced some statistical support for effects of both current (estimate = -0.1794 ± 0.1011) and early-life (estimate = -0.1430 ± 0.0716) temperature, which both suggested that individuals with lower early-life or current temperature experienced higher mortality. However, the ΔAIC values of between zero and -2 suggest that the effects were marginal.

There was no statistical support for effects of barley yield on Ikaalinen child survival, but interactions between social class and current effects of both rye and crop yields were statistically supported. These both suggested that lower and middle class individuals experienced relatively constant mortality across increasing crop yields, but that individuals of the poorest social class had high mortality when crop yields were low, but lower mortality when they increased (Figure S4). Therefore, though crop yields were associated with mortality in Ikaalinen children, but there was no interaction between early-life and current conditions in support of the predictive adaptive response hypothesis.

**Table S12: Models investigating associations between environmental variation including crop yields and Ikaalinen child mortality.**

The table presents a comparison of binomial generalised linear mixed-effects models of child mortality in Ikaalinen (aged 1–15), the only subset of the data for which sufficient data on crop yields was available to assess the effects of crops on survival. Each of the numbered models was assessed where current and early-life effects were either E, spring temperature, rye grain yield, barley grain yield, or the mean of the two grain yields. Models of each of the environmental variables were compared using AIC values. The best-supported model for each environmental variable has the lowest AIC value and is shown *in* ***bold italics***; the ΔAIC values are shown relative to the base model.

|  |  | **E** | |  | **Spring temperature** | |  | **Rye** | |  | **Barley** | |  | **Mean Crop** | |
| --- | --- | --- | --- | --- | --- | --- | --- | --- | --- | --- | --- | --- | --- | --- | --- |
| **No.** | **Model** | **AIC** | **ΔAIC** |  | **AIC** | **ΔAIC** |  | **AIC** | **ΔAIC** |  | **AIC** | **ΔAIC** |  | **AIC** | **ΔAIC** |
| 0 | BASE | 2760.45 | 0.00 |  | 2760.45 | 0.00 |  | 2760.45 | 0.00 |  | 2760.45 | 0.00 |  | 2760.45 | 0.00 |
| 1 | BASE + Current | 2759.63 | -0.82 |  | 2759.36 | -1.09 |  | 2762.11 | 1.66 |  | 2761.84 | 1.39 |  | 2761.56 | 1.11 |
| 2 | BASE + Early-life | 2762.25 | 1.80 |  | ***2758.57*** | ***-1.88*** |  | 2760.64 | 0.19 |  | 2760.06 | -0.39 |  | 2757.99 | -2.46 |
| 3 | BASE + Early-life + Current | 2761.14 | 0.69 |  | 2758.86 | -1.59 |  | 2762.54 | 2.09 |  | 2761.89 | 1.44 |  | 2759.71 | -0.74 |
| 4 | BASE + Social:Current | 2762.87 | 2.42 |  | 2762.99 | 2.54 |  | ***2753.79*** | ***-6.66*** |  | 2761.96 | 1.51 |  | ***2752.99*** | ***-7.46*** |
| 5 | BASE + Social:Early-life | 2763.80 | 3.35 |  | 2760.58 | 0.13 |  | 2762.64 | 2.19 |  | 2760.54 | 0.09 |  | 2761.06 | 0.61 |
| 6 | BASE + Age:Current | 2761.24 | 0.79 |  | 2761.29 | 0.84 |  | 2761.05 | 0.60 |  | 2763.79 | 3.34 |  | 2762.42 | 1.97 |
| 7 | BASE + Age:Early-life | 2763.48 | 3.03 |  | 2760.24 | -0.21 |  | 2759.28 | -1.17 |  | ***2759.86*** | ***-0.59*** |  | 2759.73 | -0.72 |
| 8 | BASE + Early-life:Current | ***2753.60*** | ***-6.85*** |  | 2760.70 | 0.25 |  | 2763.83 | 3.38 |  | 2762.82 | 2.37 |  | 2761.61 | 1.16 |

**Figure S3: Ikaalinen child mortality was highest where individuals experienced high early-life and current E.**

The plotted data shows that Ikaalinen child mortality was highest among individuals (aged 1 –15) who experienced high infant mortality during their year of birth and who were currently experiencing a high infant mortality year. Plot shows mean mortality across the four quartiles of current E (1 = lowest mortality, best conditions; 4 = highest mortality, worst conditions) across the four quartiles of early-life E. Points show mean mortality at each combination of current and early-life E ± 1 SE.

**Figure S4: The effect of crop yields on mortality in Ikaalinen children was strongest in the Poor social class**

The plotted data shows that Ikaalinen child mortality was only strongly affected by crop yields in the poorest social class. The best-supported models for both A) rye grain yield and B) crop grain yield contained interactions between current yield and social class. The interaction suggested that in rich individuals and middle-class individuals there was no strong association between crop yield and mortality, but in poor individuals there was higher mortality at the lowest crop yields and lower mortality at higher crop yields.

**SUPPLEMENTARY REFERENCES**

1.Dobson MJ. *Contours of Death and Disease in Early Modern England*. Cambridge: Cambridge University Press, 1997.

2.Hoch SL. Famine, disease, and mortality patterns in the parish of Borshevka, Russia, 1830-1912. *Population Studies* 1998; **52**: 357-368.

3.Turpeinen O. Infectious diseases and regional differences in Finnish death rates, 1749-1773. *Population Studies* 1978; **32**: 523-533.

4.Vihola T. Mitä ihminen tarvitsi elääkseen. In: Karonen P (ed). *Pane leipään puolet petäjäistä: Nälkä ja pulavuodet Suomen historiassa*. Jyväskylä: Jyväskylän yliopisto, 1994, 83-92.

5.Holopainen J,Helama S. Little Ice Age farming in Finland: preindustrial agriculture on the edge of the Grim Reaper’s scythe. *Human Ecology* 2009; **37**: 213-225.

6.Saarenheimo J. *Vanhan Pirkkalan Historia (History of Old Pirkkala)*. Tampere: Hameen Kirjapaino Oy, 1974.

7.Jutikkala E. The great Finnish famine, 1696-97. *Scandinavian Economic History Review* 1955; **3**: 48-63.

8.Rickard IJ, Holopainen J, Helama S *et al.* Food availability at birth limited reproductive success in historical humans. *Ecology* 2010; **91**: 3515-3525.

9.Hayward AD, Holopainen J, Pettay JE *et al.* Food and fitness: associations between crop yields and life-history traits in a longitudinally monitored pre-industrial human population. *Proceedings of the Royal Society B: Biological Sciences* 2012; **279**: 4165-4173.
